# Monte Carlo simulated correction factors for high dose rate brachytherapy postal dosimetry audit methodology

**DOI:** 10.1016/j.phro.2024.100657

**Published:** 2024-10-22

**Authors:** Krzysztof Chelminski, Alexis Dimitriadis, Roua Abdulrahim, Pavel Kazantsev, Evelyn Granizo-Roman, Jonathan Kalinowski, Shirin Abbasi Enger, Godfrey Azangwe, Mauro Carrara, Jamema Swamidas

**Affiliations:** a International Atomic Energy Agency, Department of Nuclear Sciences and Applications, Division of Human Health, Vienna, Austria; b McGill University, Department of Oncology, Medical Physics Unit, Montreal, Canada; c Lady Davis Institute for Medical Research, Jewish General Hospital, Montreal, Canada

**Keywords:** Brachytherapy, Monte Carlo simulations, Dosimetry audit, Reference Air Kerma Rate

## Abstract

**Background and Purpose:**

Full-scatter conditions in water are impractical for postal dosimetry audits in brachytherapy. This work presents a method to obtain correction factors that account for deviations from full-scatter water-equivalent conditions for a small plastic phantom.

**Material and Methods:**

A 16 × 8 × 3 cm phantom (PMMA) with a radiophotoluminescent dosimeter (RPLD) at the centre and two catheters on either side was simulated using Monte Carlo (MC) to calculate correction factors accounting for the lack of scatter, non-water equivalence of the RPLD and phantom, source model and backscatter for HDR ^60^Co and ^192^Ir sources.

**Results:**

The correction factors for non-water equivalence, lack of full scatter, and the use of PMMA were 1.062 ± 0.013, 1.059 ± 0.008 and 0.993 ± 0.009 for ^192^Ir and 1.129 ± 0.005, 1.009 ± 0.005 and 1.005 ± 0.005 for ^60^Co respectively. Water-equivalent backscatter thickness of 5 cm was found to be adequate and increasing thickness of backscatter did not have an influence on the RPLD dose. The mean photon energy in the RPLD for four HDR ^192^Ir sources was 279 ± 2 keV in full scatter conditions and 295 ± 1 keV in the audit conditions. For ^60^Co source the corresponding mean energies were 989 ± 1 keV and 1022 ± 1 keV respectively.

**Conclusions:**

Correction factors were obtained through the MC simulations for conditions deviating from TG-43, including the amount of back scatter, and the optimum audit set up. Additionally, the influence of different source models on the correction factors was negligible and demonstrates their generic applicability.

## 1 Introduction

Cervical cancer is the fourth most common cancer among women globally, whereas about 90% of the new cases and deaths worldwide in 2020 occurred in low-and middle-income countries [1]. Brachytherapy has an essential role in treating cervical cancer [2]. High Dose Rate (HDR) brachytherapy delivers the prescribed dose to the tumour at a very high dose rate of > 12 Gy/h, with a high dose per fraction (e.g., ∼7 Gy for typical fractionation schemes in cervical cancer), and if applied incorrectly can lead to under or over-dosage with the potential for adverse clinical effects. Ensuring consistent dose delivery is crucial to the quality and safety of this treatment option. This can also build public confidence in brachytherapy, which has been undermined by past reported incidents, including one fatality that was attributed to human error [3]. Dosimetry audits can prevent catastrophic incidents and minimize systematic dose variations [4], [5], [6].

Audits are not as widely available in brachytherapy as in external beam radiotherapy (EBRT), which has advanced imaging and higher automation. This could potentially render EBRT a safer option compared to brachytherapy.

The current work is part of a project aiming at developing a multilevel dosimetry audit methodology for HDR brachytherapy starting with an evaluation of the Reference Air Kerma Rate (RAKR), followed by an end-to-end audit of the entire workflow including imaging, treatment planning and delivery using clinical applicators.

The objective of this paper was to explore a phantom design for auditing the RAKR through dose measurements by:

a. determining an optimal audit set up for participating hospitals,
b. determining correction factors for conditions deviating from full-scatter water-equivalent conditions (TG-43) [7] using Monte Carlo (MC) simulations, and
c. evaluating spectral variations within the dosimeter for different ^192^Ir and ^60^Co HDR source models, with the aim to assess their influence on correction factors.

## 2 Materials and methods

### 2.1 Phantom and RPLD detector

**Fig. 1:**
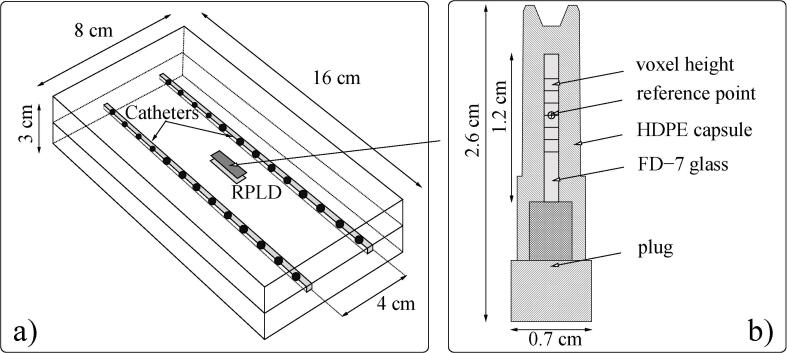
a) A schematic representation of the proposed brachytherapy dosimetry audit phantom with two channels for inserting catheters with 13 active source dwell positions indicated on either side of the RPLD and b) a larger schematic representation of the RPLD, HDPE capsule and the six voxels representing the sensitive volume used to determine the dose in MC simulations.

A simple, light-weight, cost-effective phantom was proposed suitable for remote postal dosimetry audits to assess the agreement between the RAKR of the source in afterloader and in the treatment planning. As this phantom deviates from full-scatter water-equivalent conditions, a characterization was required to determine an optimal audit setup with appropriate correction factors. A phantom with outer dimensions of 16 × 8 × 3 cm (Fig. 1a), was designed to house a radiophotoluminescent dosimeter (RPLD) at its center (Fig. 1b). The phantom had two channels on either side of the RPLD that were 15 cm in length and 2 mm in diameter to accommodate catheters of 1.7–2.0 mm diameter (5–6 Fr). The catheters were positioned symmetrically to the centre and 4 cm apart, along the long axis of the phantom. Poly Methyl Methacrylate (PMMA) was considered as a material of choice as it was cost-effective, transparent, robust, and readily available. The RPLD (Fig. 1b) was made of silver-activated phosphate glass (GD-302 M, FD-7 glass Chiyoda Technol Corporation, Japan [8]), of 1.5 mm diameter and 12 mm length, encapsulated in a watertight capsule made of high-density polyethylene (HDPE-M5001 MISATO Precision Inc., Nipolon Hard 2000, Tosoh Corporation, Japan [9]) which ensured convenient storage, transport and irradiation. The physical and chemical properties of the RPLDs used in the current study were given in Table 1 [10].

**Table 1:** Material compositions and densities of simulated phantom and structure volumes.

Material (structure)	Density (g/cm^3^)	Stochiometric formula	Atomic composition
G4_AIR	1.2 × 10^-3^	−	C(0.01%), N(75.5%), O(23.2%), A(1.3%) [16]
G4_WATER	1.00	H_2_O	H(11.2%), O(88.8%) [16]
G4_STAINLESS-STEEL (table)	8.00	−	Fe(74.0%), Cr(18.0%), Ni(8.0%) [16]
PMMA (phantom)	1.19	C_5_H_8_O_2_	C(60.0%), H(8.0%), O(32.0%) [17]
HDPE (RPLD capsule)	0.96	C_2_H_4_	C(14.4%), H(85.6%)
FD-7 (RPLD glass)	2.61	−	Ag(0.17%), Al(6.12%), Na(11.0%), P(31.55%), O(51.16%) [10]
PA12 Polyamide (catheter)	1.01	C_12_H_23_NO	C(73.0%), H(11.8%), N(7.1%), O(8.1%)

### 2.2 Treatment plan and radiation sources

**Fig. 2:**
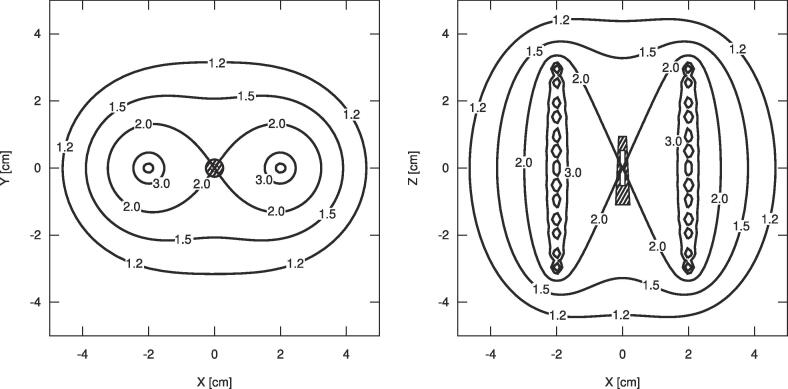
The schematic dose (Gy) distribution in the axial − XY and the coronal − XZ plane shows the 2.0 Gy – 100% isodose crossing the prescription point corresponding the location of the RPLD.

The treatment plan consisted of 13 uniform source dwell positions in each catheter with a step size of 5 mm (Fig. 1). This plan geometry was chosen to produce a homogeneous dose distribution within the RPLD volume [11]. A dose of 2 Gy was prescribed to the centre of the RPLD (Fig. 2). The dose distribution was calculated using the TG-43 formalism (SagiPlan v2.1, BEBIG Medical Germany). The planning RAKR was set at 10 mGy/h and the corresponding dwell times were 16.04 s and 16.43 s for the ^192^Ir (Bebig-GI192M11) and ^60^Co (Bebig-Co0.A86) sources respectively. The plan was exported in DICOM-RT format to perform all MC simulations [12].

### 2.3 MC simulation setup and software

The phantom, detector and backscatter material were delineated (Eclipse v15, Varian Palo Alto, USA), and exported in DICOM–RT format to RapidBrachyMCTPS [13]. The structure set, materials and densities assigned were shown in Table 1.

The Geant4 simulation toolkit (v.10.02.p02) was used through RapidBrachyMCTPS either on a local workstation or on a remote high-performance computer system (Compute Canada) [14] (Supplementary Table 1). For the dose distribution review and analysis, the Voxel Interactive Contour Tool for Online Radiation Intensity Analytics (VICTORIA) viewer was used [15]. A resolution of 1 mm in the axial direction and 1.25 mm in the sagittal and coronal directions per voxel was used for a 37 × 37 × 33 cm^3^ simulation volume. All doses were scored as the mean and the standard deviation along the 6 mm-long central axial profile through the voxels within the active volume of the RPLD (Fig. 1b).

### 2.4 Optimal audit setup

**Fig. 3:**
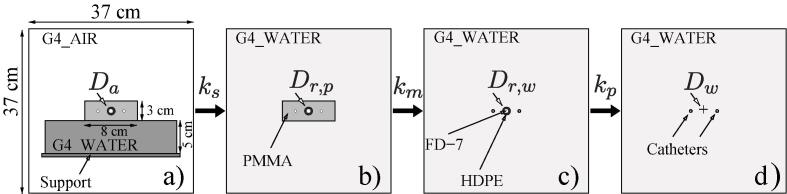
The MC simulated scenarios: a) The PMMA phantom placed on a water equivalent slab with a table underneath, b) the PMMA phantom in full-scatter water equivalent conditions, c) the RPLD and its capsule without a phantom in full-scatter water equivalent conditions, d) a water-equivalent RPLD in full-scatter water equivalent conditions.

While the proposed compact phantom might be a convenient geometry for irradiating dosimeters in a postal audit setting, the optimal setup for this phantom had not been determined. The setup options considered included placing the phantom in a water tank with near full-scatter conditions, placing the phantom on a table with partial/controlled scatter conditions, or placing the phantom “in air” with almost complete lack of scatter beyond the phantom. While placing the phantom in water would have meant that the irradiation was closer to TG-43 conditions, this setup was considered impractical, and it was discarded. The phantom “in air” option, previously used with jigs for RAKR verification with a Farmer chamber (an alternative to the use of well-type chambers still used in some countries, although not recommended [18] was also discarded as an impractical setup where the audit participant would have to place the phantom on large amounts (at least 50 cm) [19] of low-density material, such as foam, or suspend the phantom using other not standardized supports. Finally, placing the phantom on a table was considered, as it was a dry and convenient setup with the catheters and transfer tubes in near-clinical orientation. However, the amount of scatter contributions to the dose might be difficult to control due to influences from different table materials among the participants. This issue might be potentially mitigated by placing the phantom on a slab of a specific thickness and material, which was a hypothesis for investigation. A readily available material in most radiotherapy centres was water equivalent slabs, therefore, varying thicknesses (5, 10 and 15 cm) of water equivalent slabs (G4_WATER) were simulated between the phantom and the table [16]. Additionally, to simulate worst case scenarios for different tables, a low-density table represented by a 3 mm layer of air (G4_AIR) and high-density table represented by a 3 mm layer of steel (G4_STAINLESS-STEEL) were simulated under the water equivalent slabs. The setup shown in Fig. 3a with varying thickness of G4_WATER backscatter material and with high- or low-density (G4_STAINLESS-STEEL or G4_AIR) SUPPORT materials was used for these simulations.

From the simulations in auditing conditions (Fig. 3a), the following doses (*D_a_*) were extracted:

*^5l^D_a_* , *^10l^D_a_* , *^15l^D_a_* – Doses to the RPLD with water-equivalent back scatter material of 5, 10 and 15 cm thickness between the phantom and the table made of low-density (*l*-index) material of thickness of 3 mm represented by air.

*^5h^D_a_* , *^10h^D_a_* , *^15h^D_a_* – Doses to the RPLD with water-equivalent back scatter material of 5, 10 and 15 cm thickness between the phantom and the table made of high-density (*h*-index) material of thickness of 3 mm represented by steel.

### 2.5 Phantom correction factors

The correction factors for the optimal setup were obtained by simulating the four scenarios shown in Fig. 3, following the methodology proposed by Bouchard et al. [20]. Fifty million (5 × 10^6^) primary particles were simulated for 3.67–6.65 hr with two Intel Platinum 8260 Cascade Lake @2.40 GHz central processing units CPUs (Supplementary Table 1) [21]. Uncertainty of dose simulated in RPLD volume was calculated as standard deviation of dose from six adjacent voxels in the RPLD volume*.* Uncertainties of the correction factors were estimated using a differential method assessing sensitivities for uncertainties of doses [22].

Three correction factors were derived to determine the dose to water for the audit-setup:

1) *k_p_* – perturbation caused by the non-water equivalence of the RPLD and its capsule,
2) *k_m_* – non-water equivalence of the phantom material,
3) *k_s_* – lack of scatter for the phantom positioned on a table.

Detector (RPLD) perturbation correction factor *k_p_*:

$$k_{p}\equiv D_{w}/D_{r,w}$$

where, *D_w_* was the dose to a water-equivalent RPLD volume in full scatter conditions (Fig. 3d), and *D_r,w_* was the dose to the RPLD in water in full scatter conditions (Fig. 3c).

Phantom non-water equivalence correction factor *k_m_*:

$$k_{m}\equiv D_{r,w}/D_{r,p}$$

where, *D_r,p_* was the dose to the RPLD in the PMMA phantom in full scatter conditions (Fig. 3b).

Lack of scatter correction factor *k_s_*:

$$k_{s}\equiv D_{r,p}/D_{a}$$

where*, D_a_* was the dose to RPLD in the PMMA phantom placed on a water equivalent back scatter slab, in audit conditions (Fig. 3a).

Applying the correction factors, the absorbed dose to water in the RPLD could be determined:

$$D_{w}=k_{p}\cdot k_{m}\cdot k_{s}\cdot D_{a}\;.$$

### 2.6 Radiation spectrum analysis

RPLDs exhibit energy dependence [23], [24], therefore an investigation was conducted to assess the changes in the energy spectrum when using different source models. This investigation was conducted to determine if the correction factors obtained for a specific source model could be applied to measurements with other source models. The energy spectra reaching the RPLD volume were simulated for the most popular HDR source models presented in Supplementary Table 2 [25]. Two geometries were simulated for comparison:

1) the RPLD in full scatter conditions, implementing the physical properties of FD-7 for the glass and HDPE for the capsule (Table 1) at the centre of a 37 × 37 × 33 cm^3^ water cube (Fig. 3c), and
2) the proposed audit setup with the phantom on a 5 cm thick water equivalent slab with the RPLD inside the phantom (Fig. 3a).

One million (10^6^) primary particles were simulated for 7–13 min using a CPU of Lenovo ThinkCentre M720s desktop computer (Supplementary Table 1) [21]. Energy of each particle reaching the RPLD active volume was scored. The simulation was repeated five times. The mean energy of particles was calculated and assumed as an index of the radiation quality reaching RPLD. The uncertainty for the mean energy of particles was calculated using the batch method for results from five simulation runs [26].

## 3 Results

### 3.1 Optimal audit setup

The results of simulations with the phantom in all table setups were shown in the top section of Table 2. The varying thicknesses of water slabs between the phantom and the two table materials simulated did not show a significant impact on the RPLD dose with values ranging from 1.776 to 1.785 Gy (^192^Ir) and from 1.731 Gy to 1.733 Gy (^60^Co) for both air and steel with all results falling within the simulation uncertainties. The mean (± standard deviation) RPLD dose of all audit setups was *D_a_* = 1.781 ± 0.012 Gy for ^192^Ir, and *D_a_* = 1.733 ± 0.006 Gy for ^60^Co. The results suggested that a 5 cm water equivalent slab used as backscatter was sufficient to mitigate any potential impact the underlying table might have on scatter conditions, and subsequently on the dose to the RPLD.

**Table 2:** Simulated doses in Gy and correction factors for different setups for the ^192^Ir (Bebig-GI192M11) and ^60^Co (Bebig-Co0.A86 ^60^Co) sources.

	Parameter	^192^Ir	^60^Co
	*^5l^D_a_* – Dose to RPLD, 5 cm backscatter with air	1.776 ± 0.012	1.733 ± 0.006
	*^10l^D_a_* – Dose to RPLD, 10 cm back scatter with air	1.785 ± 0.011	1.731 ± 0.006
*Simulated Dose (Gy) for optimal audit setup*	*^15l^D_a_* – Dose to RPLD, 15 cm back scatter with air	1.783 ± 0.012	1.731 ± 0.006
	*^5h^D_a_* – Dose to RPLD, 5 cm backscatter with steel	1.778 ± 0.011	1.733 ± 0.006
	*^10h^D_a_* – Dose to RPLD, 10 cm backscatter with steel	1.786 ± 0.011	1.731 ± 0.006
	*^15h^D_a_* – Dose to RPLD, 15 cm backscatter with steel	1.783 ± 0.012	1.732 ± 0.006
	*D_w_* – Dose to water	1.990 ± 0.016	1.983 ± 0.006
*Simulated Dose (Gy) for correction factors*	*D_r,w_* – Dose to the RPLD in water	1.874 ± 0.016	1.757 ± 0.006
	*D_r,p_* – Dose to RPLD in PMMA phantom	1.887 ± 0.005	1.749 ± 0.006
	*D_a_* – Dose in the auditing conditions	1.782 ± 0.012	1.732 ± 0.006
	*k_p_* – Detector perturbation factor	1.062 ± 0.013	1.129 ± 0.005
*Correction factors*	*k_m_* – Phantom non-water equivalence factor	0.993 ± 0.009	1.005 ± 0.005
	*k_s_* – Lack of scatter factor	1.059 ± 0.008	1.009 ± 0.005

### 3.2 Correction factors

The doses derived from simulations and associated correction factors for the setups shown in Fig. 3 were shown in middle and lower sections of Table 2. Type A uncertainty in dose maps was calculated with the history-by-history method resulting in a mean uncertainty of 0.3% per voxel (Supplementary Table 1). The calculated uncertainty for the dose in the RPLD volume was within 0.9%. The total correction factor for the phantom setup in audit conditions (Fig. 3a), which was the product of *k_p_* , *k_m_* and *k_s_* , was 1.117 ± 0.018 for the ^192^Ir source (Bebig-GI192M11) and 1.146 ± 0.005 for the ^60^Co (Bebig-Co0.A86).

### 3.3 Radiation spectrum analysis

**Fig. 4:**
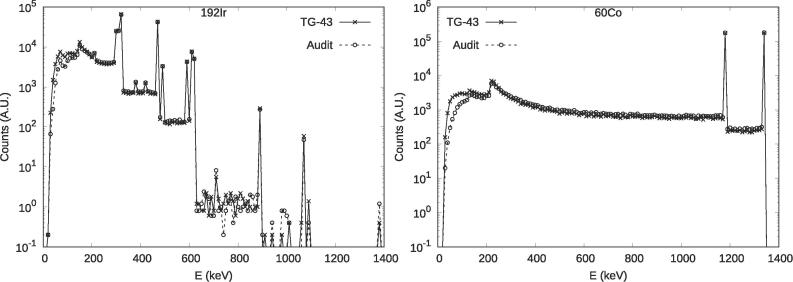
Histograms of Monte Carlo simulated particles with different energies passing through the RPLD for ^192^Ir (Bebig GI192M11) and ^60^Co (Co0.A86) HDR sources in both, the full scatter, and the audit setup. Bins of 10 keV were used.

The mean energy in the RPLD volume showed differences within one standard deviation among the ^192^Ir source models investigated in the current study. The maximum difference between the ‘full scatter’ and ‘phantom on a table’ setup was 17 keV for ^192^Ir and 34 keV for ^60^Co. The mean energy for ^192^Ir was 279 ± 2 keV and 295 ± 1 keV for the ‘full scatter’ and ‘phantom on a table’ setups, respectively. The corresponding energies for ^60^Co were 989 ± 1 keV and 1022 ± 1 keV respectively. The simulated spectra for ^192^Ir (Bebig GI192M11) and ^60^Co (Co0.A86) sources in two different geometries were shown in Fig. 4.

## 4 Discussion

The phantom, proposed for remote postal brachytherapy dosimetry audits, offers a practical and cost-effective solution. The PMMA material was chosen due to its affordability, durability, and transparency as well as its scattering properties that closely resembles water. RPLD was chosen as the detector because it is already in use for EBRT audits, making it convenient for seamless integration into the existing audit program. The “phantom on a table” with 5 cm water-equivalent material underneath was found as the optimal audit setup. The table material on which the phantom might be placed during the audit had no effect on the dose to the RPLD, with adequate backscatter material. Since, it is postal audit, the proposed phantom was envisaged to be small, lightweight, and easy to transport, that led to its deviation from TG-43 conditions. The determination of correction factors to address non-water equivalence, lack of scatter, and perturbations due to RPLD was necessary, along with the radiation spectral analysis, which was crucial in establishing a reliable dosimetry audit methodology applicable across various HDR source models being used in the hospitals.

To assess the feasibility of conducting audits in hospitals, investigation was carried out to determine the amount of backscatter needed and influence of the underlying table on the RPLD dose, on which the phantom might be positioned. The simulations demonstrated that a 5 cm thick layer of commonly used water-equivalent material, provided adequate backscatter shielding from the table regardless of the thickness and the density of the material. In some other studies 10 cm was assumed as sufficient backscatter but smaller thicknesses were not investigated [27], [28].

The G4_WATER was a representation of commercially available plastic water-equivalent materials which might differ in their scattering properties from pure water for low energy particles [29]. This question might be a basis for future simulations or experimental measurements. However, the authors do not anticipate that various commercially available water equivalent material in the hospitals would significantly impact the audit results with the proposed set-up. This expectation was based on the similarity of atomic components in these materials, the predominance of scattered radiation and existing recommendations regarding ^192^Ir dosimetry [17].

The presence of the FD-7 glass rod in a capsule made of HDPE in the PMMA phantom led to significant perturbation effects, resulting in a lower dose (6% for ^192^Ir and 13% for ^60^Co) when compared to TG-43 calculations. This might be attributed to a higher stopping power and density of FD-7 glass than water, which was corrected with the use of the detector perturbation correction factor (*k_p_*). Absorbed dose discrepancies between the TG-43 formalism and the MC simulation in non-water equivalent media was previously reported for HDR sources [30], [31].

The physical density and stopping power of PMMA compared to water resulted in 0.7% increase in dose at the detector for ^192^Ir source. Conversely, for the ^60^Co source, the dose simulated in the presence of the PMMA phantom was approximately 0.5% lower even with full scatter conditions. Commercially available water-equivalent materials used for dosimetry might help reducing this discrepancy. A phantom-material correction factor (*k_m_*) was necessary to reduce the uncertainty associated with the dose determination.

The phantom was designed to be suitable for postal audits, featuring small size and light weight for easy transport. However, this design resulted in underdosage ranging from 1% for ^60^Co to 6% for ^192^Ir. This under-dosage occurred due to the absence of scatter material around the phantom compared to TG-43 conditions and was already previously reported [32]. Submerging the phantom in a larger water tank mitigated this effect but complicated the auditing procedure. As a result, an alternative setup with an appropriate “lack of scatter” (*k_s_*) correction factor was applied to obtain dose referencing TG-43.

Deviations from the planned dose, when calculated in a TG-43 based TPS, could be accounted for by applying correction factors *k_p_* , *k_m_* and *k_s_* that were determined basing on the MC simulations from the current study. Experimental validations of the MC simulations, where possible, could support the findings of this study.

The simulations did not address the excitation or light emission response of the RPLDs caused by radiation passing through them and, hence, their innate energy dependence. However, it was important to explore how the energy spectra reaching the RPLDs differed, among the source models used in the hospitals. This was critical, as RPLDs showed an inherent energy dependence [33], [34], [35].

The simulations indicated that the HDR ^192^Ir sources were indistinguishable when considered the radiation quality criterion expressed in terms of mean particle energy reaching the detector, which was consistent with a finding of Oliver-Canamas et al. [36]. This was true for both the full scatter and audit setup geometries. However, when the geometry of measurements was changed from full scattering conditions to the audit setup, the mean energy increased by 15–17 keV for ^192^Ir and by 33–34 keV for ^60^Co. This increase in the mean energy might potentially lead to a difference in RPLD sensitivity necessitating energy correction. However, the expected difference was well below the reported uncertainty of RPLD measurements (1.6%, k = 1; [23], [24]). The most common HDR sources were simulated in this work, excluding VS2000 and the VariSource [25] as they were not available in RapidBrachyMCTPS at the time of investigation. Pulse Dose Rate (PDR) sources were also not part of this investigation. The results are therefore valid only for the investigated source models and future work may need to investigate their validity for other source models.

In the work of Hsu et al. [37], RPLDs did not exhibit energy dependence between the radiation spectra of ^192^Ir and ^60^Co HDR sources, and Hashimoto et al. [38] reported no differences between the ^192^Ir HDR source and a megavoltage photon beam from a linear accelerator at distance of 2 cm.

To conclude, the MC simulations conducted in the study determined the necessary correction factors to account for the lack of scatter and the non-water equivalence of the RPLD and phantom materials, as compared to TG-43 conditions for the HDR sources. The MC simulations identified an optimal audit setup suggesting that a 5 cm thickness of backscatter material was sufficient to mitigate the influence of the table on which the phantom might be positioned during the audits in hospitals. Moreover, the MC simulations demonstrated that the relative mean energy difference in the RPLD among various source models were negligible for both audit setup and in full-scatter conditions, which supports the use of the same correction factors for all ^192^Ir source models, simplifying the dosimetry process. Experimental validation would ensure the accuracy and reliability of the correction factors when applied to practical dosimetry audits.

## CRediT authorship contribution statement

**Krzysztof Chelminski:** Data curation, Formal analysis, Investigation, Methodology, Validation, Visualization, Writing – original draft. **Alexis Dimitriadis:** Conceptualization, Methodology, Formal analysis, Validation, Writing – original draft. **Roua Abdulrahim:** Formal analysis, Investigation. **Pavel Kazantsev:** Conceptualization, Methodology, Writing – review & editing. **Evelyn Granizo-Roman:** Software, Investigation. **Jonathan Kalinowski:** Software, Formal analysis, Writing – review & editing. **Shirin Abbasi Enger:** Resources, Software, Writing – review & editing. **Godfrey Azangwe:** Methodology, Writing – review & editing. **Mauro Carrara:** Writing – review & editing, Funding acquisition. **Jamema Swamidas:** Conceptualization, Methodology, Project administration, Resources, Funding acquisition, Supervision, Writing – review & editing.

## Declaration of competing interest

The authors declare that they have no known competing financial interests or personal relationships that could have appeared to influence the work reported in this paper.
