# Supplementary material for: Monte Carlo simulated correction factors for high dose rate brachytherapy postal dosimetry audit methodology
Source: Phys Imaging Radiat Oncol. 2024 Oct 22;32:100657. doi: 10.1016/j.phro.2024.100657 (PMC11554633; doi:10.1016/j.phro.2024.100657)
Supplement: Supplementary Data 1 [file mmc1.pdf]

Supplementary Table 1. Monte Carlo simulation information reported according to TG-268 guidelines [21].

Item name	Description	Reference
Code, version	Geant4 10.02.p02, RapidBrachyMC usercode	Agostinelli S, Allison J, Amako K, et al. GEANT4—a simula-tion toolkit. Nuclear Instrum Methods Phys Res Sect A: AccelSpectrom Detect Assoc Equip. 2003;506(3):250-303.23. DOI: 10.1016/S0168-9002(03)01368-8<br><br>Allison J,Amako K,Apostolakis J,et al. Geant4 developments and applications. IEEE Trans Nucl Sci. 2006;53(1):270-278.24. DOI: 10.1109/TNS.2006.869826<br><br>Allison J, Amako K, Apostolakis J, et al. Recent developments in Geant4. Nuclear Instrum Methods Phys Res Sect A: Accel Spectrom Detect Assoc Equip. 2016;835:186-225. DOI: 10.1016/j.nima.2016.06.125<br><br>Famulari G, Renaud M-A, Poole CM, Evans MD, Seuntjens J,Enger SA. RapidBrachyMCTPS: a Monte Carlo-based treatment planning system for brachytherapy applications. Phys Med Biol.2018;63:175007. DOI: 10.1088/1361-6560/aad97a<br><br>Glickman H, Antaki M, Deufel C, Enger SA. RapidBrachyMCTPS2.0: a comprehensive and flexible Monte Carlo-based treatment planning system for brachytherapy applications. arXiv preprintarXiv:2007.02902. 2020 Jul 6. DOI: 10.48550/arXiv.2007.02902
Timing	Dose map: 3.67 hr to 6.65 hr for 5×10^7 radioactive decays; performed on 15 threads on a node of a remote supercomputing cluster with two Intel Platinum 8260 Cascade Lake @2.40 GHz CPUs.<br><br>Spectrum simulation: 7-13 min for 10^6 radioactive decays; performed on 6 threads on Intel Core i7-8700 @3.2 GHz × 12 CPU of Lenovo ThinkCentre M720s desktop computer with 16 GB RAM and with 64-bit Linux Ubuntu 18.4.	-
Validation	Geant4/RapidBrachyMCTPS previously validated.	Famulari G, Renaud M-A, Poole CM, Evans MD, Seuntjens J,Enger SA. RapidBrachyMCTPS: a Monte Carlo-based treatment planning system for brachytherapy applications. Phys Med Biol.2018;63:175007. DOI: 10.1088/1361-6560/aad97a
Geometry	See Figure 3.	-
Materials	See Table 1.	-
Source description	Source models RapidBrachyMCTPS library, modeled from their descriptions in CLRP v2 source database. Source placement is performed according to coordinates and dwell times specified in the DICOM RTPlan. Radioactive decay is explicitly simulated using decay spectra from ENSDF.	Famulari G, Renaud M-A, Poole CM, Evans MD, Seuntjens J,Enger SA. RapidBrachyMCTPS: a Monte Carlo-based treatment planning system for brachytherapy applications. Phys Med Biol.2018;63:175007. DOI: 10.1088/1361-6560/aad97a

		Safigholi H, Chamberland MJ, Taylor RE, Martinov MP, RogersDWO, Thomson RM. Update of the CLRP Monte Carlo TG-43parameter database for high-energy brachytherapy sources. MedPhys. 2023;50(3):1928-1941. DOI: 10.1002/mp.16176<br><br>Tuli JK. Evaluated nuclear structure data file. Nuclear Instrum Methods Phys Res Sect A: Accel Spectrum Detect Assoc Equip.1996;369(2-3):506-510. DOI: 10.1016/S0168-9002(96)80040-4
Cross sections	EPDL97, EEDL97, EADL97.	Cullen DE, Hubbell JH, Kissel L. EPDL97: the evaluated photo data library '97 version. Lawrence Livermore National Lab.(LLNL), Livermore, CA (United States); 1997. DOI: 10.2172/295438<br><br>Perkins S, Cullen D, Seltzer S. Tables and Graphs of Electron-interaction Cross-sections from 10 eV to 100 GeV Derived from the LLNL Evaluated Electron Data Library (EEDL), Z=1-100. 1991;31. Lawrence Livermore National Lab: 21-24. DOI: 10.2172/5691165<br><br>Erkins S, Cullen D, Chen M, Rathkopf J, Scofield J, Hubbell J. Tables and graphs of atomic subshell and relaxation data derived from the LLNL Evaluated Atomic Data Library (EADL), Z= 1–100.UCRL-50400. U.S. Department of Energy, Office of Scientific and Technical Information; 1991. DOI: 10.2172/10121422
Transport parameters	PENELOPE low-energy electromagnetic physics list with default transport parameters. Electron transport disabled. 0.1 mm photon production cut.	Salvat F, Fernández-Varea JM, Sempau J. PENELOPE-2006: A Code System for Monte Carlo Simulation of Electron and Photon Transport. NEA No. 6222. OECD 2006;4-7. https://www.oecd-nea.org/upload/docs/application/pdf/2019-12/nea6222-penelope.pdf
Variance reduction techniques	Track length estimation enabled. Mass energy absorption coefficients taken from RapidBrachyMCTPS library.	Williamson JF. Monte Carlo evaluation of kerma at a point for photon transport problems. Med Phys. 1987;14(4):567-576. DOI: 10.1118/1.596069<br><br>Famulari G, Renaud M-A, Poole CM, Evans MD, Seuntjens J,Enger SA. RapidBrachyMCTPS: a Monte Carlo-based treatmentplanning system for brachytherapy applications. Phys Med Biol. 2018;63:175007. DOI: 10.1088/1361-6560/aad97a
Scored quantities	Collision kerma to medium in medium for the treatment plan; photon energy spectrum in the RPLD glass volume.	-
Number of histories/statistical uncertainty	5×10^7 radioactive decays per simulation, resulting in a mean uncertainty of 0.3% of dose per voxel and maximal 0.9% on the 1.7-2.0 Gy isodose line (along 6 voxels). Energy spectrum: 10^6 radioactive decays per simulation resulting in max 2 keV, (k=1) uncertainty	-
Statistical methods	Type A uncertainty in dose maps calculated with the history-by-history method. Type A uncertainty for 6 voxels along an Y isodose in RPLD.	Walters B, Kawrakow I, Rogers D. History by history statistical estimators in the BEAM code system. Med Phys.2002;29(12):2745-2752. DOI: 10.1118/1.1517611

	Uncertainties for correction factors: differential method with sensitivities for uncertainties of doses.<br>Uncertainties for energy spectrum: Type A for a batch method; standard deviation of the mean energy from 5 simulations.	BIPM I, IFCC I, ISO I, IUPAP O, eds. Evaluation of measurement data—guide to the expression of uncertainty in measurement, JCGM 100: 2008 GUM 1995 with minor corrections. Joint Committee for Guides in Metrology. Vol. 98. 2008. https://www.bipm.org/documents/20126/2071204/JCGM_100_2008_E.pdf
Postprocessing	Dose distributions visualized and analyzed in VICTORIA. Spectrum post-processing: MS Excel, Gnuplot.	Badun E, Tessier F, Townson R, Mainegra-Hing E, Storey M-A, Bazalova-Carter M. Introducing the Voxel Interactive Contour Tool for Online Radiation Intensity Analytics (VICTORIA). arXiv preprint arXiv:210514145. 2021.<br>DOI: 10.48550/arXiv.2105.14145
