# Supplementary material for: Monte Carlo simulated correction factors for high dose rate brachytherapy postal dosimetry audit methodology
Source: Phys Imaging Radiat Oncol. 2024 Oct 22;32:100657. doi: 10.1016/j.phro.2024.100657 (PMC11554633; doi:10.1016/j.phro.2024.100657)
Supplement: Supplementary Data 2 [file mmc2.pdf]

Supplementary Table 2. Characteristics of all ^{192}Ir and ^{60}Co HDR sources [25] *used in simulations. Λ is the dose-rate constant in water according to TG-43.*

Manufacturer	Source model	Radioactive isotope	Source Length (mm)	Source Diameter (mm)	Λ (cm^{-2})	Capsule material	Capsule diameter (mm)	Capsule density (g/cm^3)
BEBIG	GI192M11	^{192}Ir	3.50	0.60	1.110	316L stainless steel	1.00	7.8
BEBIG	Ir2.A85-2	^{192}Ir	3.50	0.60	1.109	316L stainless steel	0.90	7.8
Elekta	Flexisource	^{192}Ir	3.50	0.60	1.113	304 stainless steel	0.85	8.0
Nucletron	microSelectron-v1	^{192}Ir	3.50	0.60	1.116	316L stainless steel	1.10	7.8
Varian	GammaMed Plus	^{192}Ir	3.50	0.70	1.117	316L stainless steel	0.90	7.8
BEBIG	Co0.A86	^{60}Co	3.50	0.50	1.092	316L stainless steel	1.00	7.8
